# Protein Kinase A in Human Retina: Differential Localization of Cβ, Cα, RIIα, and RIIβ in Photoreceptors Highlights Non-redundancy of Protein Kinase A Subunits

**DOI:** 10.3389/fnmol.2021.782041

**Published:** 2021-11-18

**Authors:** Jinae N. Roa, Yuliang Ma, Zbigniew Mikulski, Qianlan Xu, Ronit Ilouz, Susan S. Taylor, Dorota Skowronska-Krawczyk

**Affiliations:** ^1^Department of Pharmacology, University of California, San Diego, La Jolla, CA, United States; ^2^Microscopy and Histology Core Facility, La Jolla Institute for Immunology, La Jolla, CA, United States; ^3^Department of Physiology and Biophysics and Department of Ophthalmology, Center for Translational Vision Research, University of California, Irvine, Irvine, CA, United States; ^4^The Azrieli Faculty of Medicine, Bar Ilan University, Safed, Israel; ^5^Department of Chemistry and Biochemistry, University of California, San Diego, La Jolla, CA, United States

**Keywords:** PKA, retina, mitochondria, photoreceptors, neuron, signaling

## Abstract

Protein kinase A (PKA) signaling is essential for numerous processes but the subcellular localization of specific PKA regulatory (R) and catalytic (C) subunits has yet to be explored comprehensively. Additionally, the localization of the Cβ subunit has never been spatially mapped in any tissue even though ∼50% of PKA signaling in neuronal tissues is thought to be mediated by Cβ. Here we used human retina with its highly specialized neurons as a window into PKA signaling in the brain and characterized localization of PKA Cα, Cβ, RIIα, and RIIβ subunits. We found that each subunit presented a distinct localization pattern. Cα and Cβ were localized in all cell layers (photoreceptors, interneurons, retinal ganglion cells), while RIIα and RIIβ were selectively enriched in photoreceptor cells where both showed distinct patterns of co-localization with Cα but not Cβ. Only Cα was observed in photoreceptor outer segments and at the base of the connecting cilium. Cβ in turn, was highly enriched in mitochondria and was especially prominent in the ellipsoid of cone cells. Further investigation of Cβ using RNA BaseScope technology showed that two Cβ splice variants (Cβ4 and Cβ4ab) likely code for the mitochondrial Cβ proteins. Overall, our data indicates that PKA Cα, Cβ, RIIα, and RIIβ subunits are differentially localized and are likely functionally non-redundant in the human retina. Furthermore, Cβ is potentially important for mitochondrial-associated neurodegenerative diseases previously linked to PKA dysfunction.

## Introduction

Vision is the most appreciated of the five senses, and age- and disease-related loss of visual perception has devastating impacts on quality of life. Several studies with mouse retina degeneration models implicate the second messenger cyclic adenosine 3′,5′ monophosphate (cAMP) and cAMP-dependent protein kinase A (PKA). Tight regulation of intracellular cAMP levels is critical in the modulation of photoreceptor light adaptation (Cohen and Blazynski, 1990; Cohen et al., 1992; Nir et al., 2002) and rod outer segment shedding and renewal (Stenkamp et al., 1994), and recent studies using PKAchu mice expressing Förster resonance energy transfer (FRET)-based PKA activity sensor protein, suggest a role for PKA in dark adaptation in rods (Sato et al., 2020). PKA signaling at mitochondria is also important for fission/fusion and mitophagy (Dagda and Banerjee, 2015), both of which are essential for maintaining high rates of oxidative phosphorylation in the photoreactive zones of the retina.

**FIGURE 1:**
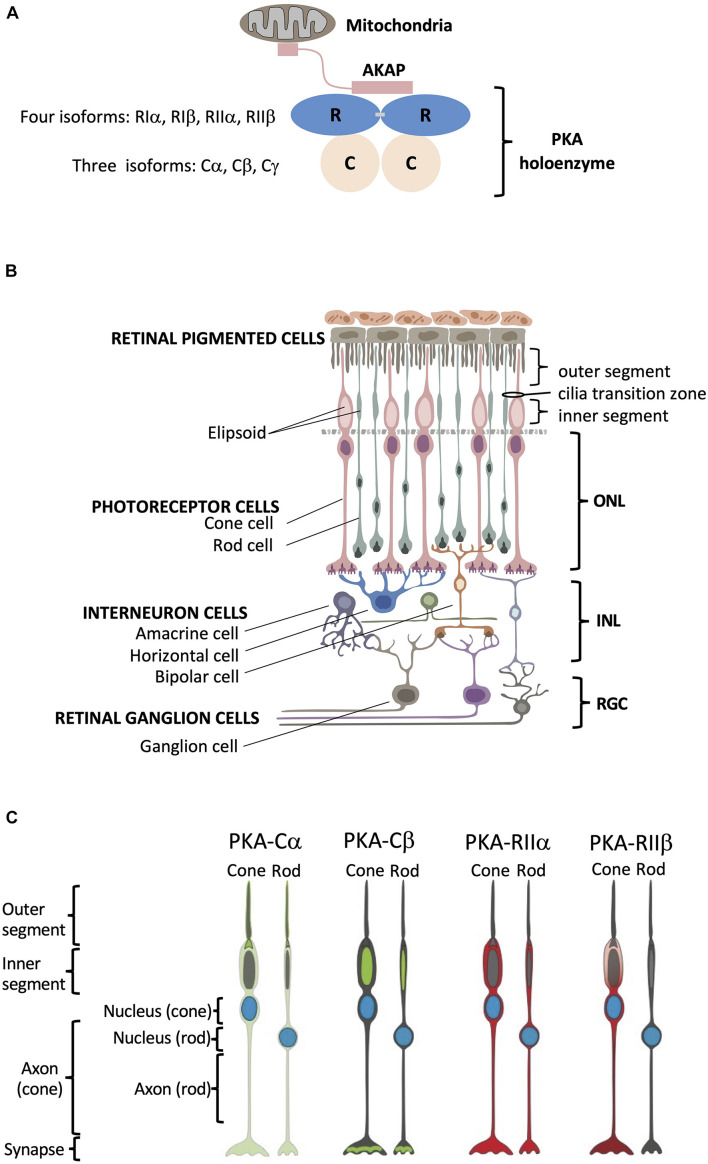
Structural organization of PKA and human retina. **(A)** Schematic of PKA. In the inactive state two catalytic subunits are bound by a regulatory subunit dimer, which is typically anchored to intracellular locations such as the mitochondria by AKAP proteins. **(B)** Retina consists of six neuronal cell types: cone, rod, horizontal, bipolar, amacrine, and ganglion cells. These six cell types are organized into three main layers within the retina that include the outer nuclear layer (ONL) with the nuclei of cones and rods, the inner nuclear layer (INL) with nuclei of interneuron horizontal, bipolar, and amacrine cells, and the retinal ganglion cell layer (RGC) that contains ganglion cell nuclei. Photoreceptor cone and rod cells directly accept photons in the specialized part of the cell called the outer segment, and they contain a mitochondrion-enriched ellipsoid in the inner segment, with the area connecting the outer and inner segment referred to as the cilia transition zone. RPE cells (top) are phagocytosis-competent cells indispensable for daily outer segment renewal. For interneurons: horizontal cells communicate with photoreceptors; bipolar cells communicate with both photoreceptor and retinal ganglion cells; and amacrine cells communicate with retinal ganglion cells. Retinal ganglion cells convey the visual information to the brain. **(C)** Summary of PKA localization in cone and rod photoreceptor cells. Cα: outer segment membrane, base of the connecting cilium, cell body, axon, and synapse of cone and rod cells. Cβ: inner segment mitochondrion-enriched ellipsoid of cone and rod cells, as well as in the synaptic region. RIIα: cell body, axon, and synapse of cone and rod cells. RIIβ: cell body, axon, and synapse of cone cells.

Eukaryotic cells express multiple forms of PKA regulatory (R) and catalytic (C) subunits, and this subunit diversity accounts in large part for PKA functional specificity. In general, PKA holoenzymes consist of an R subunit dimer bound to two C subunits (R_2_C_2_) (Figure 1A). The biochemical and functional features of PKA holoenzymes are largely determined by the structure and the biochemical properties of the four functionally non-redundant regulatory subunits, RIα, RIβ, RIIα and RIIβ (McKnight et al., 1988; Ilouz et al., 2012; Taylor et al., 2012; Zhang et al., 2012). Spatially restricted localization of these holoenzymes, mediated primarily by scaffold proteins referred to as A Kinase Anchoring proteins (AKAPs), provides an important layer of specificity in PKA signaling (Michel and Scott, 2002) while the unique cell-type specific and subcellular of RIβ and RIIβ in brain supports their functional non-redundancy (Ilouz et al., 2017). Although current dogma emphasizes the importance of subcellular localization of AKAP-bound R subunits in the functional diversity of PKA signaling (Pawson and Scott, 2010), little attention has been paid to the subunit diversity of the C subunits, especially in the brain, the only tissue where many of the Cβ splice variants are expressed at high levels. While Cα is ubiquitously expressed in all mammalian cells, RNA *in situ* hybridization suggests 50% of PKA signaling in brain is due to Cβ (Skalhegg and Tasken, 2000; Ørstavik et al., 2001). Without spatial information on endogenous protein localization, however, it is difficult to hypothesize how these two C subunits might differentially influence regulation of specific neuronal processes and to provide evidence for their functional non-redundancy.

Here we used the human retina with its highly specialized neurons as a model system to explore subcellular localization of PKA, which will serve as a window into PKA signaling in the brain. The retina, a highly organized and experimentally accessible part of the central nervous system, is composed of three distinct cell layers with six easily distinguishable neuronal cell types (Figure 1B). Using specific antibodies raised against Cα, Cβ, RIIα, and RIIβ, as well as other cell-type specific and organelle markers, we detailed the spatial localization of PKA C and RII subunits in tissue sections of human retina and found distinct localization patterns for each PKA subunit. The use of human samples gives us the opportunity to investigate the details of human retinal biology rather than using animal models, such as mice, which do not faithfully recapitulate all specifics of human retina structure and disease. Using this system, we show that (1) Cβ is associated primarily with mitochondria, (2) Cα is the only subunit of the four that is enriched in the photoreceptor outer segment cell membrane and at the base of connecting cilium, and (3) Cα, RIIα, and RIIβ are all excluded from mitochondria-rich inner segment ellipsoid but still show distinct localization patterns in photoreceptors (Figure 1C). In addition, using novel RNA BaseScope technology, we achieved the first insights into the cell-type specific distribution of the Cβ splice variants in neurons.

## Results

### PKA C and RII Subunits Are Differentially Localized in Retina

**FIGURE 2:**
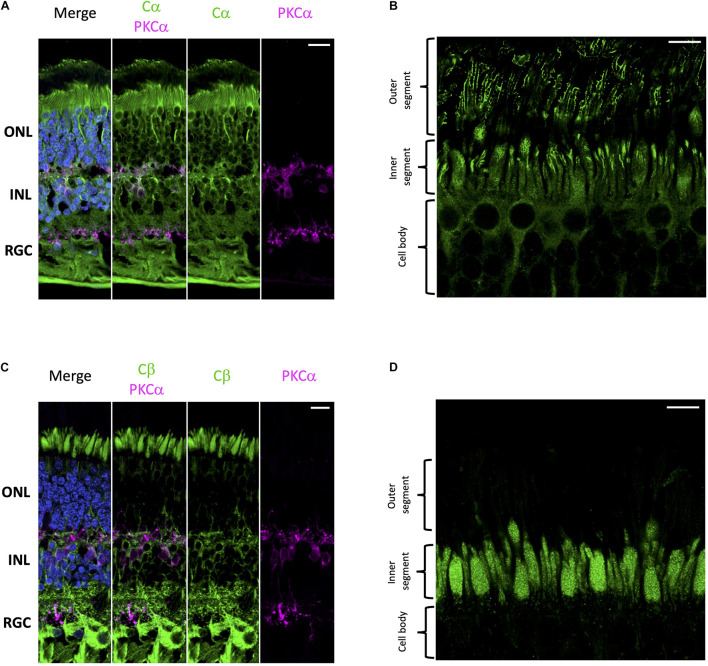
Cα and Cβ are differentially localized in human retina. **(A–D)** All sections were labeled with anti-PKCα antibodies in order to identify rod-bipolar cells (purple), which helped distinguish between the outer nuclear layer (ONL), inner nuclear layer (INL), and retinal ganglion cell layer (RGC). Nuclei in blue, scale bar = 20 μm **(A,C)**, 10 μm **(B,D)**. **(A)** Cα (green) is localized to cells in the ONL, INL, and RGC. **(B)** In photoreceptors, Cα is generally localized to the cell body of both cone and rod photoreceptor cells; however, there is increased signal at the junction between the inner and outer segment. **(C)** Cβ (green) is localized to cells in the ONL, INL, and RGC. **(D)** In photoreceptors, Cβ (green) was limited to the ellipsoid of the inner segment, and notably absent from the outer segment and the junction connecting the inner and outer segment.

Using well-established antibodies against PKA C and RII subunits we stained retinal cryosections from human donor samples. To visually distinguish the different cell layers of the retina we used PKCα as a standard marker for rod bipolar cell synapses. Our results show that Cα is diffusely distributed throughout the cell body of all cell types in the retina (Figure 2A); however, in photoreceptors, Cα is also present in the outer segment plasma membrane and in the region that connects the inner and outer segment. Notably, however, Cα did not localize with the photoreactive pigments (rod and cone opsins); therefore, it is not present in the photoreceptor disks (Figure 2B and Supplementary Figure 1). Strikingly, Cβ localization is non-overlapping with that of Cα. Cβ is highly enriched in photoreceptors, interneurons, and retinal ganglion cells (Figure 2C). Strikingly, Cβ is localized to the photoreceptor inner segment ellipsoid; however, it is not present in the photoreceptor outer segment or in the region connecting the inner and outer segment (Figure 2D and Supplementary Figure 1). The striking differences in the subcellular localization of Cα and Cβ strongly suggests that these subunits carry out non-redundant physiological functions.

**FIGURE 3:**
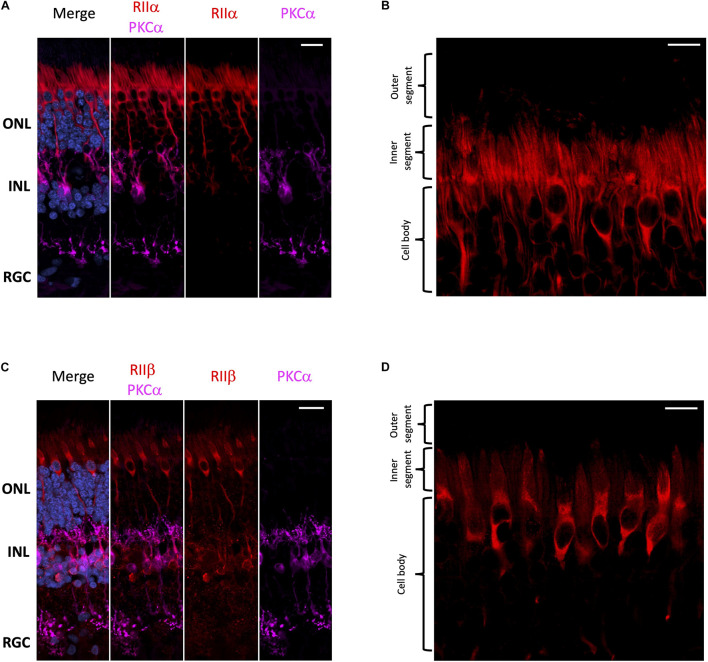
RIIα and RIIβ are differentially localized in human retina. **(A–D)** All sections were labeled with anti-PKCα antibodies in order to identify rod-bipolar cells (purple), which helped distinguish between the outer nuclear layer (ONL), inner nuclear layer (INL), and retinal ganglion cell layer (RGC). Nuclei in blue, scale bar = 20 μm **(A,C)**, 10 μm **(B,D)**. **(A)** RIIα (red) is localized to cells in the ONL and INL, but not the RGC. **(B)** In photoreceptors, RIIα (red) is generally localized to the cell body and axons of both cone and rod photoreceptor cells. **(C)** RIIβ (red) is localized to cells in the ONL and INL, but not the RGC. **(D)** In photoreceptors, RIIβ (red) localization was distinct and strongest in the cell body and axons of cone photoreceptors.

With respect to regulatory subunits, RIIα and RIIβ are both localized to photoreceptors and interneurons (Figure 3); but they show distinct subcellular localization patterns (Figure 3). RIIα is clearly enriched in both rod and cone cell body and axons (Figures 3A,B), while RIIβ appears to be selectively enriched in cone cells in regions that surround the nucleus (Figures 3C,D). It is important to note that neither RIIα nor RIIβ are localized to the photoreceptor outer segment, the region connecting the inner and outer segment, or the inner segment ellipsoid. Remarkably, we did not observe strong localization for RIIα and RIIβ in retinal ganglion cells even though both Cα and Cβ are present in these cells.

### PKA C and RII Subunits Show Distinct Patterns of Co-localization

**FIGURE 4:**
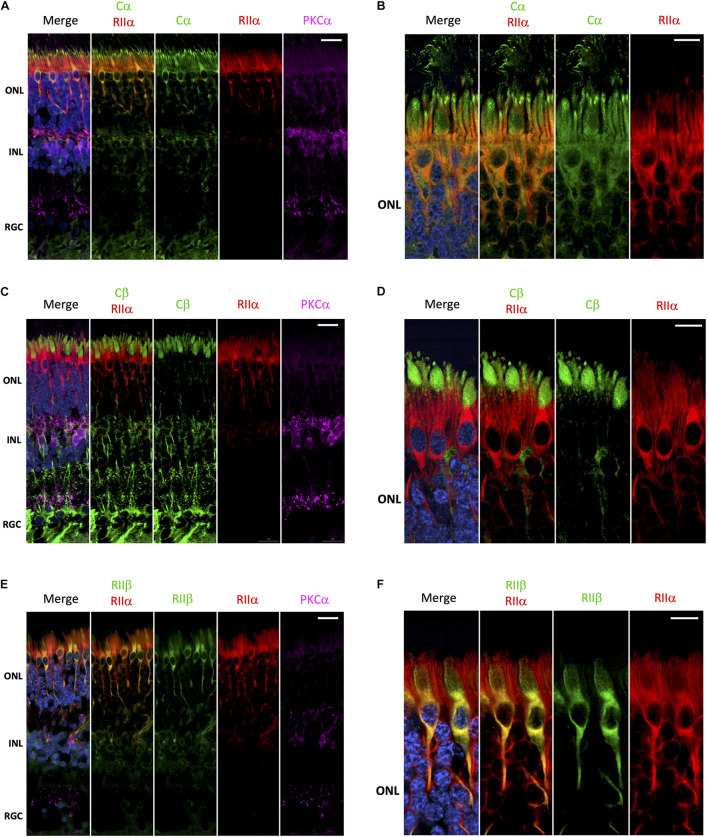
Cα, but not Cβ, co-localizes with RIIα; RIIα and RIIβ show similar localization. **(A–F)** All sections were labeled with anti-PKCα antibodies in order to identify rod-bipolar cells (purple), which helped distinguish between the outer nuclear layer (ONL), inner nuclear layer (INL), and retinal ganglion cell layer (RGC). Nuclei in blue, scale bar = 20 μm **(A,C,E)**, 10μm **(B,D,F)**. **(A)** Cα (green) and RIIα (red) co-localized in photoreceptors and interneurons in the ONL and INL (orange, Cα RIIα). Only Cα was localized to the RGC. **(B)** In photoreceptors, Cα (green) and RIIα (red) clearly co-localized in the cell body and axons (orange, Cα RIIα); however, only Cα was present in the outer segment membrane and at the junction connecting the inner and outer segment. **(C)** Cβ (green) and RIIα (red) intracellular localization differs in cells in the ONL and INL, with no signal overlap (Cβ RIIα) in photoreceptors or interneurons. Only Cβ was localized to the RGC. **(D)** Cβ (green) and RIIα (red) both clearly localized to photoreceptors; but Cβ is found in the inner segment, while RIIα is found in the cell body and axons, with no signal overlap (Cβ RIIα). **(E)** RIIβ (green) and RIIα (red) co-localized in cells in the ONL and INL, with strong signal overlap (orange, RIIβ RIIα) in photoreceptors and interneurons. Neither RIIβ and RIIα were localized to the RGC. **(F)** In photoreceptors, RIIβ (green) and RIIα (red) clearly co-localized to the cell body and axons (orange, RIIβ RIIα); however, RIIβ was distinctly enriched in cone photoreceptor cells, while RIIα was diffusely distributed in both rod and cone photoreceptor cells.

Surprisingly, it is has not been rigorously established whether there is a preference for PKA-R subunits to form holoenzymes with Cα, Cβ or even potentially Cα/Cβ heterotetramers. Furthermore, it is unknown whether C-subunit composition of a particular holoenzyme varies from one cell type to another. To begin to address these questions, we performed a series of co-stainings to deduce the potential composition of PKA holoenzymes in the various retina cell types. Cα and RIIα are clearly co-localized in photoreceptor and interneuron cells (Figure 4A), which would be consistent with these subunits existing as a holoenzyme complex. In photoreceptors, this co-localization is restricted to the cell body, with no co-localization between RIIα and Cα in the region connecting the inner and outer segment (Figure 4B and Supplementary Figure 2). In contrast to Cα, Cβ is not co-localized with RIIα; although both seem to be expressed in photoreceptors and interneurons (Figure 4C). Subcellular localization in photoreceptors further confirms these differences, with localization of Cβ limited to the inner segment and RIIα distributed throughout the cell body and axon (Figure 4D and Supplementary Figure 3). Finally, RIIα and RIIβ are co-localized in photoreceptor and interneuron cells (Figure 4E); however, RIIβ was more prevalent in cone cells, making co-localization of the two subunits stronger in this cell type, specifically in areas surrounding the nucleus (Figure 4F and Supplementary Figure 4).

### Cβ Is Localized With Mitochondria

**FIGURE 5:**
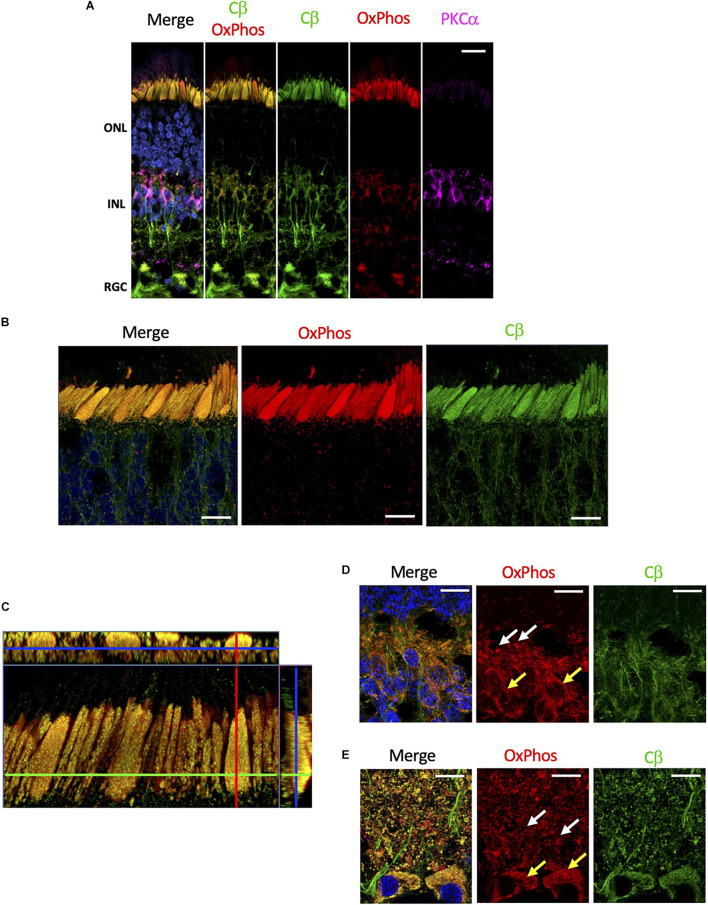
Cβ is co-localized with mitochondria. **(A)** All sections were labeled with anti-PKCα antibodies in order to identify rod-bipolar cells (purple), which helped distinguish between the outer nuclear layer (ONL), inner nuclear layer (INL), and retinal ganglion cell layer (RGC). Cβ (green) and OxPhos(red)-labeled mitochondria co-localize in the ONL, INL, and RGC, with clear signal overlap (yellow, Cβ OxPhos) in every cell layer. **(B)** In photoreceptors, Cβ (green) and OxPhos(red)-labeled mitochondria are distinctly co-localized to the inner segment ellipsoid (yellow, merge). Additionally, Cβ-positive mitochondria puncta can be visualized below the inner segment ellipsoid. **(C)** Cβ and OxPhos-labeled mitochondria co-localize (yellow) to fill the entire inner segment ellipsoid of a single cone cell (outlined). **(D,E)** Cβ (green) and mitochondria (red) are clearly co-localized (yellow) in photoreceptor synapses **(D)** and retinal ganglion cells **(E)**. Mitochondria localization: white arrows – plexiform layer, yellow arrows – soma. Nuclei in blue, scale bar = 10 μm.

Although mitochondria are present in every cell, they are enriched in rod and cones cells. These cells have a substantial biosynthetic burden as proteins and lipids in the outer segment are replaced every day (Kevany and Palczewski, 2010), and expend a significant amount of energy during the light/dark cycle to supply the ATP required to maintain ion transport at synaptic terminals (Ingram et al., 2020). Given the selective localization of Cβ to the mitochondrion-rich inner segment ellipsoid, we wanted to specifically investigate if Cβ co-localizes with mitochondria. To achieve this, we used anti-OxPhos antibodies created against mitochondrial encoded cytochrome C oxidase 1 (MT-CO1) located in the mitochondrial inner membrane. Our results show that Cβ does co-localize with mitochondria in all cell layers (Figure 5A and Supplementary Figure 5), which is best visualized in photoreceptors where the signal from anti-Cβ antibodies completely overlapped with mitochondria identified using anti-OxPhos antibodies (Figure 5B and Supplementary Figure 5). This was further confirmed using multiple z-stack images that show co-localization in the ellipsoid was complete, which we visualized in a single compiled z-stack cross section as well as throughout nine individual z-stack images (Figure 5C and Supplementary Figure 6).

Interestingly, Cβ colocalized with mitochondria also in other regions. In photoreceptors, we noticed OxPhos-positive small mitochondria below the ellipsoid, in the neurites and in the outer plexiform layer which co-localized with Cβ signal. Additionally, we observed Cβ puncta colocalizing with mitochondria in the synapses in the inner plexiform layer (Figure 5D). Finally, we detected high level of Cβ in RGCs where it also clearly localized with two populations of mitochondria – in the soma and in the axons of RGCs (Figure 5E). Notably, significant amounts of Cβ did not co-localize with mitochondria across the retina, seemingly located in the elongated structures suggestive of axonal ER (Supplementary Figure 5). This staining is very pronounced in the nerve fiber layer where axons of RGCs are bundled together.

### Cα Is Localized at the Base of the Connecting Cilium and on the Outer Segment Plasma Membrane

**FIGURE 6:**
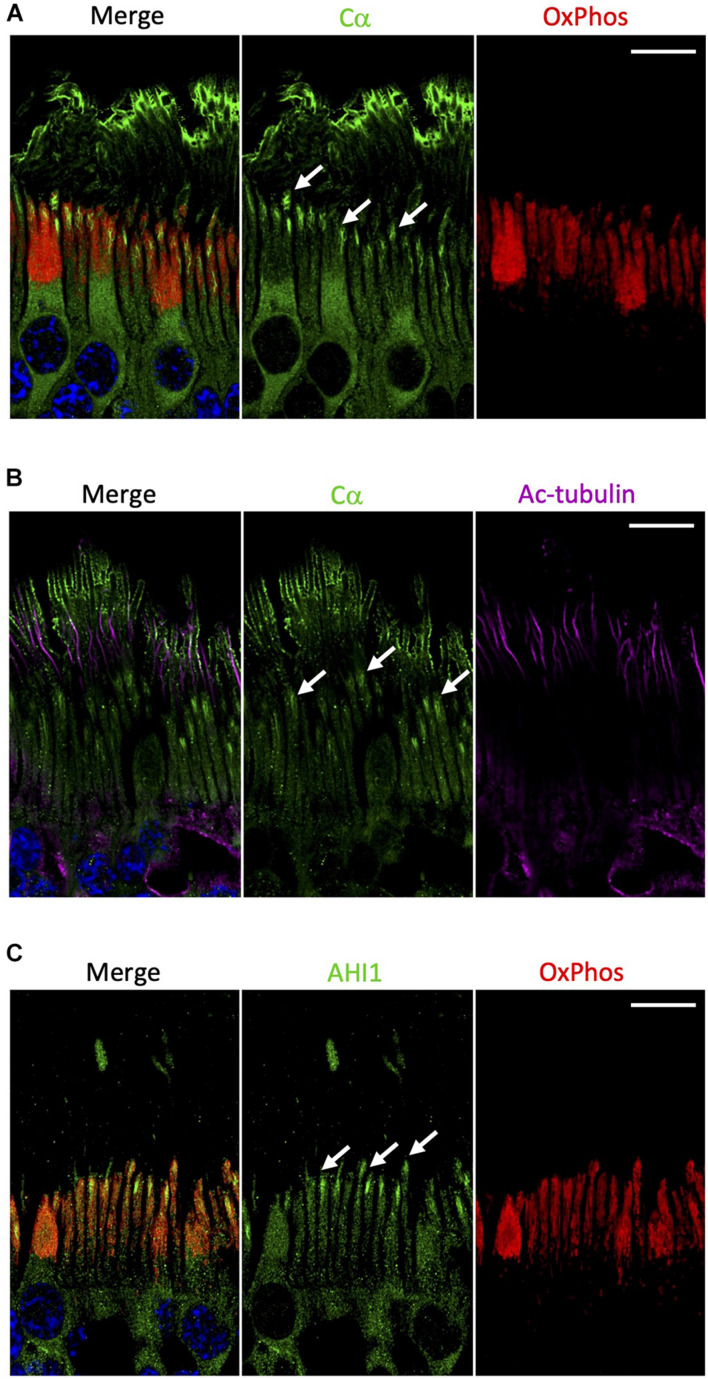
Cα is not localized with mitochondria but is localized at the base of the connecting cilium. **(A)** In photoreceptors, Cα (green) was distinctly separate from OxPhos (red)-labeled mitochondria. Cα signal was strongest in the cell body, at the base of the connecting cilium, and on the outer segment plasma membrane. OxPhos signal was enriched in the mitochondria-containing ellipsoid of the photoreceptor inner segment. **(B)** Cα (green) and acetylated tubulin (purple) did not co-localize in photoreceptor cells, but instead showed stacked localization with acetylated tubulin in the axoneme above Cα at the base of the connecting cilium. **(C)** Similar to Cα, AHI1 (green) was localized at the base of the connecting cilium in the region connecting the inner and outer segment. **(A–C)** Arrows indicate localization at the base of the connecting cilium. Nuclei in blue, scale bar = 10 μm.

In contrast to Cβ, our results show that Cα was notably absent from the mitochondrion-rich ellipsoid of the inner segment (Figure 6A and Supplementary Figure 7). Although Cα is constitutively expressed in all cells and distributed diffusely throughout the cell body and in the inner segment of photoreceptor cells, there are other regions in the rod and cone cells where Cα is exclusively enriched (Figure 2). Given that the outer segment is the highly specialized cilium stemming from the inner segment, we wanted to investigate the potential co-localization of Cα with the elements of this region. To do that we used antibodies against acetylated tubulin, a marker of the photoreceptor cilia axoneme including the connecting cilium (Robichaux et al., 2019), and antibodies against AHI1 (Abelson helper integration site 1), which is a protein localized to the base of the connecting cilium (Louie et al., 2010) that acts as a barrier to prevent diffusion of transmembrane proteins. Our results show a lack of co-localization of Cα and acetylated tubulin (Figure 6B), which reveals Cα detected in the outer segment is not localized to the axoneme and is suggestive of cell-membrane localization of Cα in the photoreceptor outer segment. In contrast, AHI1 localization to the space connecting the inner and outer segment was strikingly similar to Cα localization in the inner segment (Figure 6C). Although the AHI1 and Cα antibodies cannot be used together as they were both raised in rabbit, the comparison of Cα/OxPhos (Figure 6A) and AHI1/OxPhos (Figure 6C) images confirms Cα localization to the base of the connecting cilium.

### Cβ Splice Variants Are Differentially Expressed Across the Retina

**FIGURE 7:**
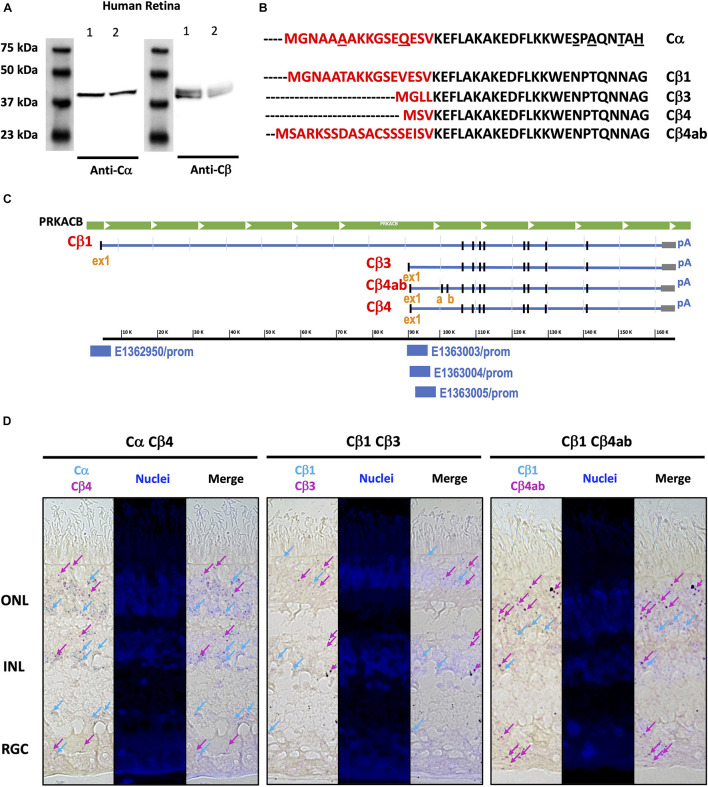
Similar Cα and Cβ proteins are present but multiple Cβ splice variants show variable expression across the human retina. **(A)** Anti-Cα antibodies recognized a ∼39 kDa band, and anti-Cβ antibodies recognized two bands at ∼39 and ∼42 kDa bands, in Western blots from two samples (Cohen and Blazynski, 1990; Cohen et al., 1992) of human retina tissue extracts. **(B)** Protein sequence alignment of Cα and Cβ splice variants Cβ1, Cβ3, Cβ4, and Cβ4ab, and **(C)** Corresponding RNA sequence alignments of Cβ isoforms, with unique promoter sequences identified for each variant explored. ex1, exon 1; a, b, exon a, exon b. **(D)** BaseScope Duplex assay results show Cα, Cβ1, Cβ3, Cβ4, and Cβ4ab expression in photoreceptors in the outer nuclear layer (ONL) and interneurons in the inner nuclear layer (INL). Cβ4 and Cβ4ab were also expressed in the retinal ganglion cell layer (RGC). Expression of Cα and Cβ1 highlighted by blue arrows, and expression of Cβ3, Cβ4, and Cβ4ab highlighted by magenta arrows.

Western blots of human retina samples show two bands for Cβ, while only one band is present for Cα (Figure 7A). These results suggest (or “are consistent with”) the presence of multiple Cβ isoforms in human retina, a finding that is further supported by a previous RNA hybridization study that revealed tissue-specific expression of multiple Cβ isoforms (Ørstavik et al., 2001). As seen on the protein sequence alignments of Cα and the multiple Cβ isoforms, the variants in Cβ are due exclusively to differences in the N-terminus (Figure 7B; Skalhegg and Tasken, 2000; Ørstavik et al., 2001; Taylor et al., 2021). Since, with the exception of Cβ2, they all have approximately the same molecular weight, they cannot be distinguished by gel migration nor with the current available antibodies that recognize antigens common to all Cβ isoforms. The Cβ antibody that we use, for example, was generated against an epitope in the C terminal tail (Asp324) that is shared by all Cβ isoforms. Because of the latter, immunohistochemistry cannot presently distinguish between different Cβ isoforms.

To overcome limitations of existing antibody-based reagents, we turned to RNAscope technology to define the localization of various Cβ isoforms to different cell types. When examining the Cβ locus we noticed that each variant has a unique exon one, further confirmed by the ENCODE *in silico* prediction of promoter sequences (ENCODE Project Consortium et al., 2020) coinciding with each first exon, and with additional splice variants that shuffle short exons (4β vs. 4βab) (Figure 7C). RNA sequence alignments detected differences in Cβ1, Cβ3, Cβ4, and Cβ4ab at the N-terminus that were sufficient to design BaseScope probes specific for each isoform (Supplementary Figure 8). Our *in situ* hybridization results show that Cα is expressed in all retinal cell layers, which is consistent with previous findings that Cα is ubiquitously expressed in the body. Interestingly, Cβ isoforms displayed more distinctive expression patterns. While Cβ4 and Cβ4ab are expressed in all retinal cell types, Cβ1 seems to be less abundant and Cβ3 is practically excluded from RGCs (Figure 7D and Supplementary Figure 9).

## Discussion

In this study we investigated the subcellular localization of PKA catalytic (C) and regulatory (R) subunits in human retinal samples, whose finely differentiated and spatially organized neurons serve as an experimentally accessible “window into the brain.” Our study highlights the importance of using human tissues to investigate issues of human health and disease, especially with regard to the visual system. Human tissues are understandably extremely difficult to obtain and model species are a great tool to study a wide range of processes; however, there are several fundamental characteristics that define the human visual system that make human tissues the best option for its study. By using human tissues, we eliminated the ambiguity that often comes from attempting to apply data from a model species to human health. By exploring localization of endogenous proteins in human tissues, our study serves as a valuable companion to structural analysis of signaling proteins like PKA and traditional exploration of protein function using various immortalized cell lines.

### Cβ in Mitochondria

Both Cα and Cβ subunits seem to be expressed in all neurons; however, we show striking co-localization of Cβ with mitochondria, while Cα appears to be excluded from this organelle. Localization of Cβ to mitochondria was confirmed by co-staining sections with anti-Cβ antibodies and antibodies against cytochrome *c* oxidase, the terminal complex of the electron transfer chain. Mitochondrial localization of Cβ is most noticeable in the large ellipsoid body of cone cells due to the presence of densely packed mitochondria, while in other cells such as the retinal ganglia cells, the mitochondria and Cβ are still co-localized but are distributed throughout the cytoplasm. Additional enrichment of Cβ was detected in mitochondria localized to synapses, RGC somas, and RGC axons further emphasizing the Cβ/mitochondria co-localization in retinal cells.

It has been previously shown that PKA is an essential regulator of different aspects of mitochondrial biology (Feliciello et al., 2005). Multiple lines of evidence show the pivotal role of PKA in mitochondrial morphology, dynamics, and turnover by phosphorylating dynamin-related protein 1 (Drp1) to inhibit mitochondrial fission (Strack et al., 2013; Edwards et al., 2020) the proapoptotic protein BAD to promote survival (Virdee et al., 2000), and mitochondrial-anchored cytoskeletal proteins to remodel dendrites (Dagda and Banerjee, 2015). PKA phosphorylation also has important roles within mitochondria that include phosphorylation of a subunit of complex I (NDUFS4) *in vitro* and *in vivo* to elevate oxidative phosphorylation (DeRasmo et al., 2008), and phosphorylation of subunits of complex I and IV during metabolically generated pH stress (Acin-Perez et al., 2009). Our data suggest that these functions are most likely subserved by the Cβ subunit of PKA, pinpointing the non-redundant roles of both Cα and Cβ. Further studies are required to understand which Cβ isoform variant is involved in specific mitochondrial processes and where in the mitochondria the Cβ subunit is localized. Our imaging shows that Cβ is densely packed in the ellipsoid of cone cells but we do not distinguish whether localization is at the outer membrane, inner membrane, or inner membrane space. Going forward it will also be important to investigate the role of Cβ in human disease with specific regards to PKA signaling in mitochondria, which has been linked to several neurodegenerative diseases including Alzheimer’s, Huntington’s, and Parkinson’s Disease (Dagda and Banerjee, 2015).

### Cβ Splice Variants

Given that the Cβ splice variants are so similar in sequence, differing only at the region corresponding to the first exon, it is not possible to discriminate between the Cβ family members with currently available antibodies. As a first step to decipher whether the Cβ protein variants are expressed in a cell-specific manner and whether they represent a set of functionally non-redundant proteins, we used RNA BaseScope technology, which allows for the detection of small differences in the RNA transcript. This novel approach allowed us to demonstrate that the Cβ variants are expressed in a cell-specific manner. In particular, while Cβ1 and Cβ3 are expressed in photoreceptors and interneurons, Cβ1 is particularly enriched in these cells. In contrast, while the Cβ4 and Cβ4ab are expressed in most retinal cells, they appear to be the primary Cβ isoforms expressed in RGCs. This suggests that specific Cβ protein variants have cell-type specific roles and that their distribution may indicate particular cellular functions. It is also important to note here that while most of known PKA disease mutations are associated with either Cα (Cushing’s Disease) or RIα (Carney Complex Disease or Acrodysostosis), a recent report described patients with four different Cβ mutations who all showed a severe and complex phenotype that includes skeletal and cardiac defects as well as a polydactyl presentation (Palencia-Campos et al., 2020). These pathological mutations in Cβ also inhibit Sonic hedgehog (Shh) signaling. This raises the possibility that some of the critical roles of Cβ in the central nervous system including the eye, is to regulate neurodevelopment and cilia biology through Shh pathways.

### Cα in Photoreceptor Cells

Interestingly, of all the subunits explored in this study, Cα was the only one found to be highly enriched in the photoreceptor outer segment plasma membrane and at the base of the connecting cilium. Regarding the outer segment membrane, this sequestration of a PKA C subunit to the cell membrane could be related to the recent report showing that the PKA C subunit is directly bound in a non-canonical way to Smoothened, a GPCR that is linked to the Sonic hedgehog pathway and is localized to cilia (Arveseth et al., 2021). Although there is still no evidence of Smoothened in the outer segment of photoreceptors, a recent study has shown that Gli1 (an effector of Smoothened) is present in the outer segment (Gupta et al., 2018). Strikingly, the same work shows the localization of Smoothened is at the base of the connecting cilium, similar to the Cα data we present in this work. Additionally, Cα localized at the tip of the outer segment membrane is in close proximity to the retinal pigment epithelium (RPE) cells. RPE cells are responsible for removal and recycling of outer segment material daily (Kevany and Palczewski, 2010), and with the supposed high mitochondrial turnover rate in photoreceptor cells it is possible that the PKA pathway could play a role in photoreceptor regeneration via RPE cells. It is important to note again that Cα was not localized to the inner membrane stacks of the outer segment that house the photoreactive pigments (Supplementary Figure 1); however, because of its proximity in the plasma membrane, it is possible that PKA activity still has some downstream effects on the photoreactive pigments and on the visual cycle in general. In fact, a recent study suggests that dark adaptation in photoreceptor cells is linked to PKA as it acts as a substrate for PKG signaling in cultured 661W cells (Roy et al., 2021), a cone-like cell that serves as a proxy for studies focused on photoreceptor cells.

Regarding the connecting cilium, we used co-staining with acetylated tubulin and parallel staining with AHI1 to corroborate our initial discovery of Cα localized at the junction of the inner and outer segment. Our results confirmed Cα is localized to the base of the connecting cilium. Cα did not co-localize with acetylated tubulin and was therefore not in the axoneme of the connecting cilium (Robichaux et al., 2019); however, and in contrast, Cα was localized similarly to AHI1, which was previously shown to be present at the base of the connecting cilium within the transition zone of the mouse retina (Louie et al., 2010). The enrichment of Cα in this region is especially interesting because it provides a novel opportunity to study the disease mechanisms of Usher syndrome, a complicated disease previously linked to the cAMP pathway that is defined by several mutations of cilia-associated proteins (Kremer et al., 2006; Mathur and Yang, 2015), as well as other blinding diseases that arise from improper regulation of the outer segment sensory cilium (Wensel et al., 2016). Additionally, Cα localized to this area could potentially play a role in photoreceptor intracellular communication as the base of the connecting cilia, also considered the cilia transition zone, essentially acts as a funnel where vesicle trafficking between the inner and outer segments is highly regulated (Gilliam et al., 2012; Wensel et al., 2021).

### RIIα vs. RIIβ

With respect to R subunits, this study focused on the RII class of regulatory subunits that include RIIα and RIIβ. Unlike the pseudo-substrate nature of the RI subunits, RII subunits both inhibit activity of and are phosphorylated by the C subunits (Taylor et al., 2012). In addition, the RII subunits are mostly targeted through AKAPs to receptors, ion channels, and transporters placing them near their target substrates. Early small angle X-ray scattering studies (Vigil et al., 2004, 2006) and the recent cryoEM structure of the RIIβ holoenzyme suggest that the two RII isoforms also have distinct conformations (Lu et al., 2020). Our results further support previously reported by selective KO studies, the functional non-redundancy of RIIα and RIIβ, as we show distinct, but overlapping, localization patterns for each. While both are highly enriched in the photoreceptor cells, RIIα is clearly present in both rods and cones while RIIβ is especially high in cones, which suggests that RIIα provides some additional light/dark related regulation in rod cells. Interestingly, RIIβ localization in cones displayed distinct enrichment around the nucleus where Golgi and ER are typically enriched.

At this point we were not able to explore the localization of RIα and RIβ. Based on our preliminary data with mouse retina we predict both subunits are present in retinal cells; however, the conditions for immunostaining with antibodies for detecting RIα and RIβ in human tissues still need to be optimized. This is now a major focus for the next phase of our research of PKA in retina. Interestingly, our preliminary data suggest RIβ may be the predominant subunit localized to retinal ganglion cells (Roa et al., in preparation), which would explain why in the current study we did not observe regulatory subunits in retinal ganglion cells or colocalized with Cβ.

## Conclusion and Future Perspectives

While localization of functionally non-redundant PKA holoenzymes and R subunits by AKAPs has been widely recognized as a way of achieving specificity in PKA signaling, the role of the C subunit in this process has been largely ignored. Early hybridization studies showed decades ago that the Cβ subunit constitutes over half of the PKA-C in the brain. However, little attention was paid to the specific localization and functional roles of the Cβ proteins which include several sub families as well as multiple splice variants. Our results showing intra- and inter-cellular differences of Cα/Cβ and RIIα/RIIβ localization provide a basis for functional non-redundancy of PKA in rod and cone cells with a direct relevance to human health. Furthermore, our demonstration of distinct patterns of localization in the highly specialized neurons of the retina suggests that this is a rich and diverse world of PKA signaling that has yet to be explored.

## Materials and Methods

### Patient Information

Human retina tissue sections and retinas extracts were obtained from normal (age 81, 83, and 91 years) donors (San Diego Eye Bank, CA, United States) with appropriate consent from the San Diego Eye bank and with a protocol approved by the University of California, San Diego Human Research Protection Program. Donors have no history of eye disease, diabetes, or chronic central nervous system disease. One donor contributed retina tissue sections and corresponding localization data, and two donors contributed retina extracts to obtain data presented in Western blots.

### Tissue Processing

After enucleation, eyeballs were fixed for ∼24 h in the 10% formalin. Next, the anterior segment, crystalline lens and vitreous were removed, and the eye cups were processed for cryostat sections (12 μm). Sections were stored long-term at –80°C.

Frozen sections were defrosted (10 min, RT) and placed in 1X PBS (10 min, RT). Rehydrated sections were then blocked with 2% normal donkey serum, 0.02% keyhole limpet hemocyanin in 1X PBS-TX (0.2% Triton-X) for 1h. Sections were then incubated in primary antibodies overnight at 4°C. Slides were then washed in 1X PBS (3x, 15 min, RT) and sections were incubated in DAPI and secondary antibodies (2 h, RT). Slides were then washed in 1X PBS (3x, 15 min, RT) and sections were permanently mounted in Fluorogel with tris buffer (Electron Microscopy Sciences, Hatfield, PA, United States).

### Antibodies

Primary antibodies were generally used at 1:100 dilution, including: custom rabbit serum anti-Cα antibodies; rabbit polyclonal anti-Cβ antibodies (Lifespan Biosciences, catalog # LS-C191947); mouse monoclonal anti-RIIα antibodies (Santa Cruz Biotechnology, catalog # sc-137220, RRID: AB_2268608); rabbit monoclonal anti-RIIβ antibodies (Abcam, catalog # AB75993, RRID: AB_1524201); and AF647 conjugated anti-PKCα antibodies (Santa Cruz Biotechnology, catalog # sc-8393, RRID: AB_628142). Custom mouse monoclonal anti-RHO (1D4 anti-rhodopsin) antibodies were used at 1:1000, and mouse monoclonal anti-OxPhos antibodies (Complex IV, Subunit I, Thermo Fisher Scientific, catalog # 459600, RRID: AB_2532240) were used at 1:500. Peanut Agglutinin conjugated with rhodamine was used at 1:1000 to identify cones (Vector Laboratories, catalog # RL-1072-5). Mouse anti-acetylated tubulin antibodies (Zymed, catalog # 6-11B-1) and custom rabbit anti-AHI1 antibodies (Louie et al., 2010) were used at 1:250. Secondary antibodies: Alexa Fluor 488 Donkey anti-rabbit (Jackson ImmunoResearch Laboratories, RRID: AB_2313584) and Alexa Flour 594 Donkey anti-mouse (Jackson ImmunoResearch Laboratories, RRID: AB_2338871) were used at 1:250. Nuclei were stained with DAPI (2 μg/ml). Custom anti-Cα antibodies specifically recognized purified Cα protein at ∼39 kDa but not purified Cβ protein, while pan anti-Cβ recognized purified Cβ protein at ∼39 kDa but not purified Cα protein (Supplementary Figure 10); and sections incubated with secondary antibodies as negative controls showed no signal (Supplementary Figure 10).

### Imaging

High resolution images were captured using a laser scanning confocal mode (A1R HD, Nikon) on an Eclipse Ti2-E (Nikon) housed in the UCSD Nikon Imaging Center. Samples were excited with 405,488, 561, and 640 nm laser from a laser unit (LU-NV, Nikon) and emission captured using a slowed galvano scanner mode. Images were either acquired at 40x (S Fluor 40x NA 1.30 oil) or 100x (Plan Apo lambda 100x NA 1.45 oil). 40x images were used to determine general protein localization patterns, and 100x images were used to determine cell- and organelle-specific protein localization. Cα, Cβ, RIIα, RIIβ, OxPhos, RHO, and PNA image acquisition details: Laser Power 3.0%, High Voltage 10–25, Offset 5, LUT range 100–3000. PKCα image acquisition details: Laser Power 4.5%, High Voltage 90–100, Offset 0, LUT range 50–2000. DAPI image acquisition details: Laser Power 5.0%, High Voltage 60, Offset 5, LUT range 60–745. Pixel size was set to 170 nm, and pixel dwell time was > 1 μs with unidirectional scan mode. Digital images were adjusted for brightness and contrast only, using Omero Insight software (University of Dundee & Open Microscopy Environment). Antibody specificity was verified on control sections incubated with only secondary antibodies and similarly processed, including image acquisition parameters and post-processing in Omero Insight.

To obtain 3D images of the tissue, additional high-resolution images were taken using a ZEISS LSM880 with Airyscan at the La Jolla Institute for Immunology. Samples were excited with 405, 488, and 561 nm laser light with main beam splitters set to 405 nm, and 488/561/633 nm. Laser power was kept below 5%. Images were acquired with Plan-Apochromat 40x NA 1.4, Plan-Apochromat 63x NA 1.4, or Alpha Plan-Apo 63x NA 1.46 oil objectives using Immersol 518F 30°C immersion oil. For imaging DAPI, emitted fluorescence was filtered by a bandpass 420-480 nm + longpass 605 nm filter. Detection of Alexa Fluor 488 utilized a bandpass 420-480 + 495-550 nm filter, and fluorescence of Alexa Fluor 568 was collected with 570 nm longpass filter and a bandpass 420–480 + 495–620 nm filter. Pixel size was set to 40 nm, and pixel dwell time was > 1 μs with unidirectional scan mode. Z-stacks were acquired with optimal step size according to ZEN Black 2.3 SP1 software. Airyscan detector was automatically aligned and run in the super-resolution mode with gain settings between 700 and 750 V to achieve optimal dynamic range. Data were processed with automatic Airyscan processing settings and resulting 16-bit images were adjusted for brightness and contrast using ZEN Blue 3.1 lite software. Maximum intensity or orthogonal projections were created for selected images.

### Western Blots

Retina tissues were lysed with RIPA buffer to get the whole cell lysate. Fifty μg of each sample was separated on a 10% gel and blotted for probing with anti-Cα and Cβ, respectively. Thirty ng of purified Cα and Cβ was also included in the gel as specific control.

### BaseScope Duplex Assay

BaseScope duplex assay for the mRNA detection was performed following the manual of BaseScope duplex detection kits (ACD, 323870) to detect splicing variants of PKA Cβ within which, four of them bare a difference with only a few nucleotides. Fresh frozen sections were rinsed in PBS and baked at 60°C for 1 h followed by dehydration in 50, 75 and 100% ethanol. Formaldehyde-fixed paraffin-embedded (FFPE) sections were 60°C baked and deparaffinized twice in xylene and twice in ethanol. Sections were dried and treated with hydrogen peroxide for 10 min, then the samples were heated in targets retrieval buffer at 99°C for 15 min followed by incubation for 3 min in ethanol. Protease treatment was performed using protease III at 40°C for 30min. Sections were washed in water and the target mRNA were hybridized with probes by incubation at 40°C for 2 h. Probes were designed and produced by Advanced Cell Diagnostics. Sections were washed in wash buffer followed by a cascade of hybridization steps with signal amplification molecules and added green and red substrates to the targets. Both the steps for Amp7 and Amp11 in BaseScope duplex detection kits manual were prolonged to 45 h. After all the hybridization steps, nuclei in the sections were stained with DAPI, dehydrated with xylene, and mounted with VectaMount medium (Vector Labs).

Probes used in the assay:

**Cα1** (NM_002730.4): BA-Hs-PRKACA-3zz-st, Cat#868751, Lot#20164A
**Cβ1** (NM_002731.3):BA-Hs-PRKACB-tv2-2zz-st, Cat# 867501, Lot#20164A
**Cβ3** (NM_001242858.2): BA-Hs-PRKACB-tv5-2zz-st-C2, Cat#868741, Lot#20164A
**Cβ4** (NM_001375565.1): BA-Hs-PRKACB-tv18-1zz-st-C2, Cat#867391-C2, Lot#20164A
**Cβ4ab** (NM_001375560.1):BA-Hs-PRKACB-tv13-1zz-st-C2, Cat#868731-C2, Lot#20164A.

## Data Availability Statement

The original contributions presented in the study are included in the article/Supplementary Material, further inquiries can be directed to the corresponding author/s.

## Ethics Statement

Ethical review and approval was not required for the study on human participants in accordance with the local legislation and institutional requirements. Written informed consent for participation was not required for this study in accordance with the national legislation and the institutional requirements.

## Author Contributions

ST and DS-K conceived the project, guided experimental design, and interpretation of the data. RI began to explore retina as a model system. RI and YM preformed original exploratory experiments. JR conducted the all IHC experiments for the manuscript and Nikon imaging. ZM conducted the Airyscan imaging. YM conducted the western blot experiments. YM and QX conducted the BaseScope experiments. JR prepared all the figures for the manuscript. JR, ST, and DS-K wrote the manuscript. All the authors read and edited the manuscript.

## Conflict of Interest

The authors declare that the research was conducted in the absence of any commercial or financial relationships that could be construed as a potential conflict of interest.

## Publisher’s Note

All claims expressed in this article are solely those of the authors and do not necessarily represent those of their affiliated organizations, or those of the publisher, the editors and the reviewers. Any product that may be evaluated in this article, or claim that may be made by its manufacturer, is not guaranteed or endorsed by the publisher.

## Supplementary Material

Supplementary Figure 1 Airyscan images of Cα and Cβ localization in cones and rod cells. Sections stained with anti-Cα or anti-Cβ with anti-RHO (rod cell marker) or anti-PNA (cone cell marker) antibodies highlight intracellular differences in Cα and Cβ localization in rod and cone cells, respectively. **(A)** Cα (green) is in the cell body, cilia transition zone, and outer segment membrane, and is distinctly separate from anti-RHO (red) in the outer segment intracellular space. **(B)** Cβ (green) is inner segment ellipsoid and is distinctly separate from anti-RHO (red) in the outer segment intracellular space. **(C)** Cα (green) is in the cell body, cilia transition zone, and outer segment membrane, and is distinctly separate from anti-PNA (red) in the outer segment intracellular space. **(D)** Cβ (green) is inner segment ellipsoid and is distinctly separate from anti-PNA (red) in the outer segment intracellular space.

Supplementary Figure 2 Airyscan images of Cα + RIIα in photoreceptor cells. Z-stack Airyscan images confirm intracellular localization of Cα (green, Cα) and RIIα (red, RIIα), with clear co-localization of Cα and RIIα (yellow, Merge) in the photoreceptor cell body and axon. Only Cα is expressed at the base of the connecting cilium and outer segment membrane. Nuclei in blue, scale bar = 10 mm.

Supplementary Figure 3 Airyscan images of Cβ + RIIα in photoreceptor cells. Z-stack Airyscan images confirm intracellular localization of Cβ (green, Cβ) and RIIα (red, RIIα), with distinctly different localization of Cβ and RIIα (Merge). Only Cβ is expressed in inner segment ellipsoid, while RIIα is localized to photoreceptor cell body and axon. Nuclei in blue, scale bar = 10 mm.

Supplementary Figure 4 Airyscan images of RIIβ + RIIα in photoreceptor cells. Z-stack Airyscan images confirm intracellular localization of RIIβ (green, RIIβ) and RIIα (red, RIIα), with clear co-localization of RIIβ and RIIα (yellow, Merge) in the cell body and axon of cone cells. Nuclei in blue, scale bar = 10 mm.

Supplementary Figure 5 Airyscan images of Cβ + mitochondria. Cb (green) and OxPhos(red)-labeled mitochondria co-localize in the ONL, INL, and RGC, with clear signal overlap (yellow) in every cell layer. Nuclei in blue, scale bar = 10 mm.

Supplementary Figure 6 Z-stack images of Cβ + mitochondria. Nine z-stack Airyscan images highlight intracellular localization of Cβ and mitochondria co-localization (yellow). As the panels move from image 1 (top stack, blue line) to image 9 (bottom stack, blue line) it is clear that Cβ is continuously co-localized mitochondria in throughout the entire photoreceptor inner segment. Blue line = z position (primary image), green line = top panel, red line = right panel. Green and red lines highlight localization in an individual cell, which can be further visualized in the top and right panel.

Supplementary Figure 7 Z-stack images of Cα + mitochondria. Nine z-stack Airyscan images highlight intracellular localization of Cα (green) with respect to mitochondria (red). As the panels move from image 1 (bottom stack, blue line) to image 9 (top stack, blue line) it is clear that Cα is continuously expressed in the outer segment membrane. There is clear signal from Cα (green) at the top of the outer segment in images 1–3, in the mid region in images 4–6, and near the base (closer to the inner segment mitochondria ellipsoid) in images 7–9. Images 1–9 also confirm Cα (green) is not present in the mitochondria ellipsoid (red). Blue line = z position (primary image), green line = top panel, red line = right panel. Green and red lines highlight localization in an individual cell, which can be further visualized in the top and right panel.

Supplementary Figure 8 RNA sequence alignments for Cβ1, Cβ3, Cβ4, and Cβ4ab. Differences at the N-terminus were sufficient to design BaseScope probes specific for each isoform (red section), with sequence similarities continuing beyond exon 1 (green section).

Supplementary Figure 9 BaseScope Duplex assay of Cα, Cβ1, Cβ3, Cβ4, and Cβ4ab expression. All isoforms are expressed in photoreceptor and interneuron cells, with Cβ4 and Cβ4ab prominently expressed in all tissue layers. Color deconvolution was done following the technical note of ACD (TS 46-003/RevA/Date 6212018). The green color and red color were separated with Image J, darkened, and merged together using Adobe Photoshop.

Supplementary Figure 10 Antibody control for Cα and Cβ. **(A)** Anti-Cα antibodies specifically recognized purified Cα protein and not purified Cβ4 protein in Western blots, while anti-Cβ antibodies recognized purified Cα protein and not purified Cβ4 protein. Both Cα and Cβ4 were present at the predicted size of ∼39 kDa. **(B)** Human retina sections used as negative controls for corresponding IHC data showed no detectable signal after incubation with secondary donkey anti-rabbit and donkey anti-mouse antibodies.
