# Supplementary figures and images for: Protein Kinase A in Human Retina: Differential Localization of Cβ, Cα, RIIα, and RIIβ in Photoreceptors Highlights Non-redundancy of Protein Kinase A Subunits
Source: Front Mol Neurosci. 2021 Nov 18;14:782041. doi: 10.3389/fnmol.2021.782041 (PMC8636463; doi:10.3389/fnmol.2021.782041)

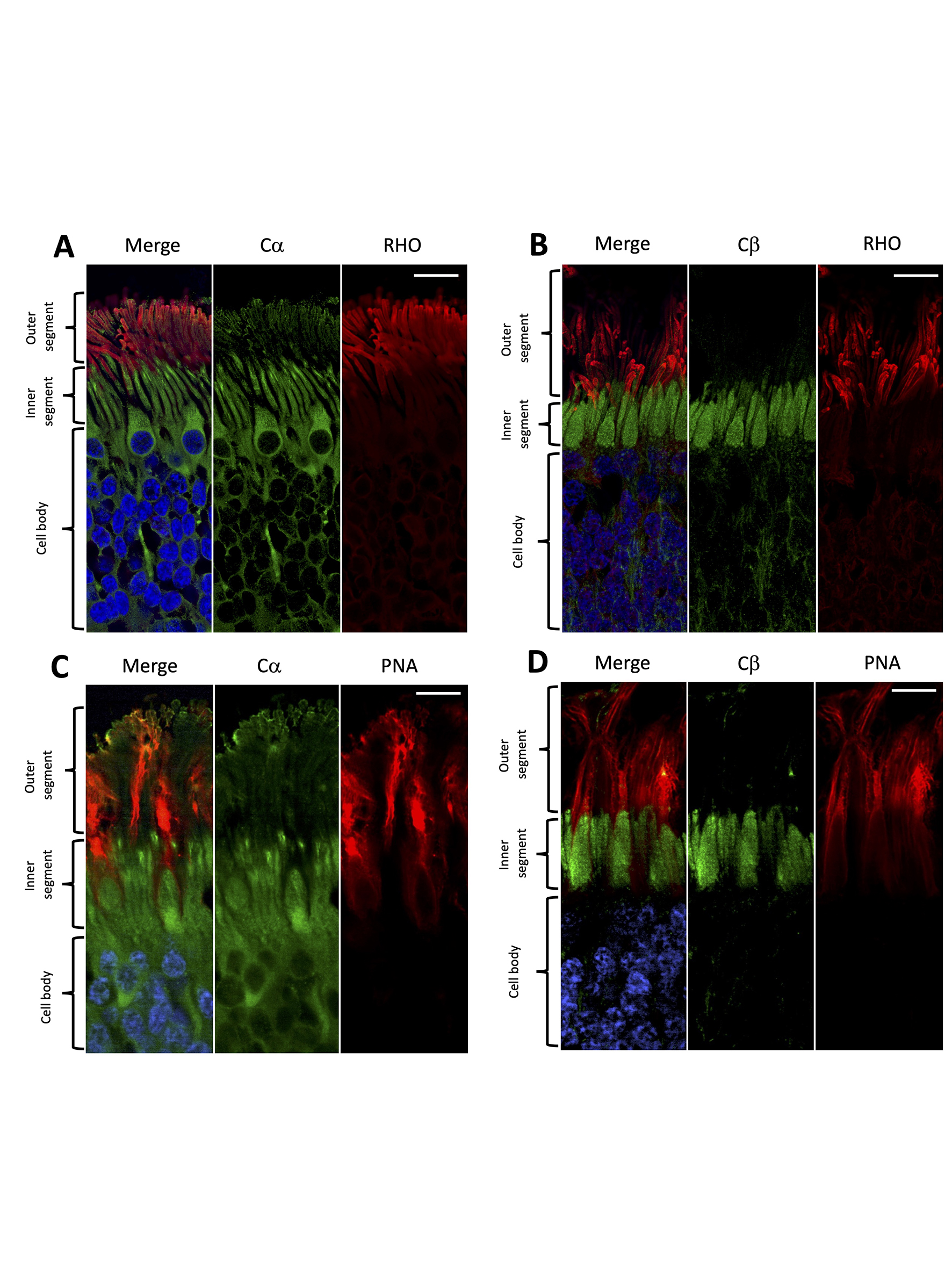

Supplement: Supplementary Figure 1 — Airyscan images of Cα and Cβ localization in cones and rod cells. Sections stained with anti-Cα or anti-Cβ with anti-RHO (rod cell marker) or anti-PNA (cone cell marker) antibodies highlight intracellular differences in Cα and Cβ localization in rod and cone cells, respectively. (A) Cα (green) is in the cell body, cilia transition zone, and outer segment membrane, and is distinctly separate from anti-RHO (red) in the outer segment intracellular space. (B) Cβ (green) is inner segment ellipsoid and is distinctly separate from anti-RHO (red) in the outer segment intracellular space. (C) Cα (green) is in the cell body, cilia transition zone, and outer segment membrane, and is distinctly separate from anti-PNA (red) in the outer segment intracellular space. (D) Cβ (green) is inner segment ellipsoid and is distinctly separate from anti-PNA (red) in the outer segment intracellular space. [file Image_1.JPEG]

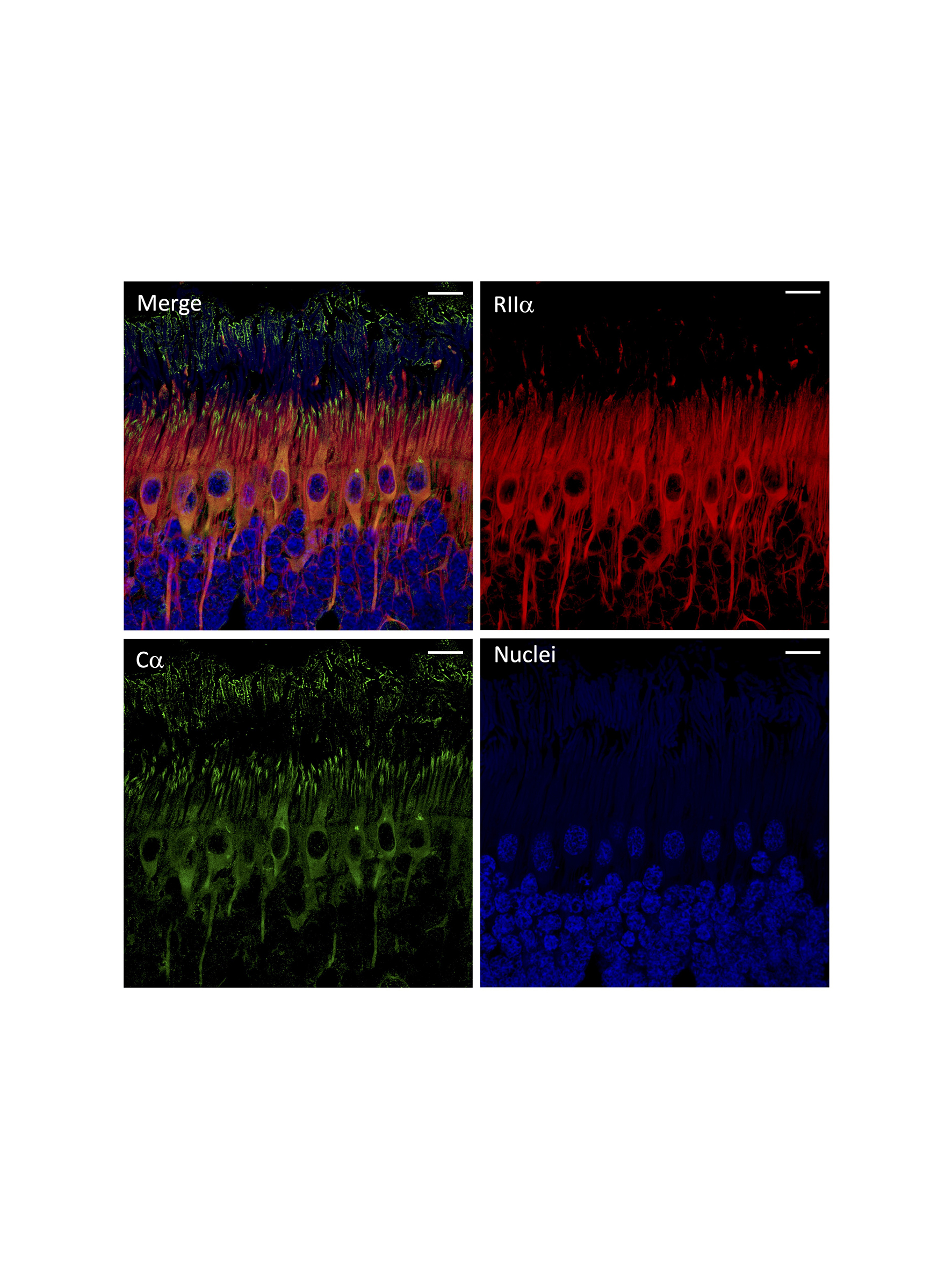

Supplement: Supplementary Figure 2 — Airyscan images of Cα + RIIα in photoreceptor cells. Z-stack Airyscan images confirm intracellular localization of Cα (green, Cα) and RIIα (red, RIIα), with clear co-localization of Cα and RIIα (yellow, Merge) in the photoreceptor cell body and axon. Only Cα is expressed at the base of the connecting cilium and outer segment membrane. Nuclei in blue, scale bar = 10 mm. [file Image_2.JPEG]

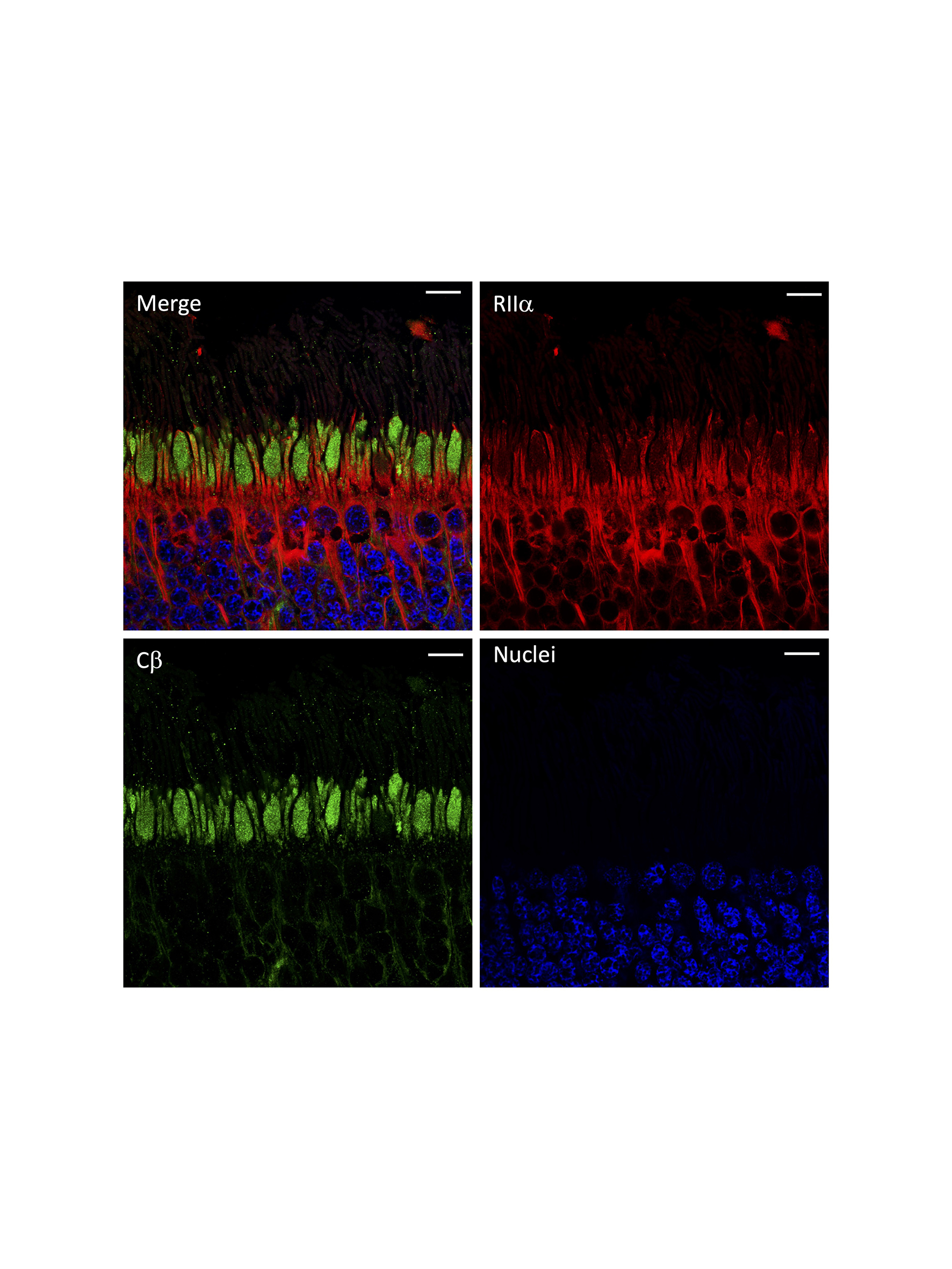

Supplement: Supplementary Figure 3 — Airyscan images of Cβ + RIIα in photoreceptor cells. Z-stack Airyscan images confirm intracellular localization of Cβ (green, Cβ) and RIIα (red, RIIα), with distinctly different localization of Cβ and RIIα (Merge). Only Cβ is expressed in inner segment ellipsoid, while RIIα is localized to photoreceptor cell body and axon. Nuclei in blue, scale bar = 10 mm. [file Image_3.JPEG]

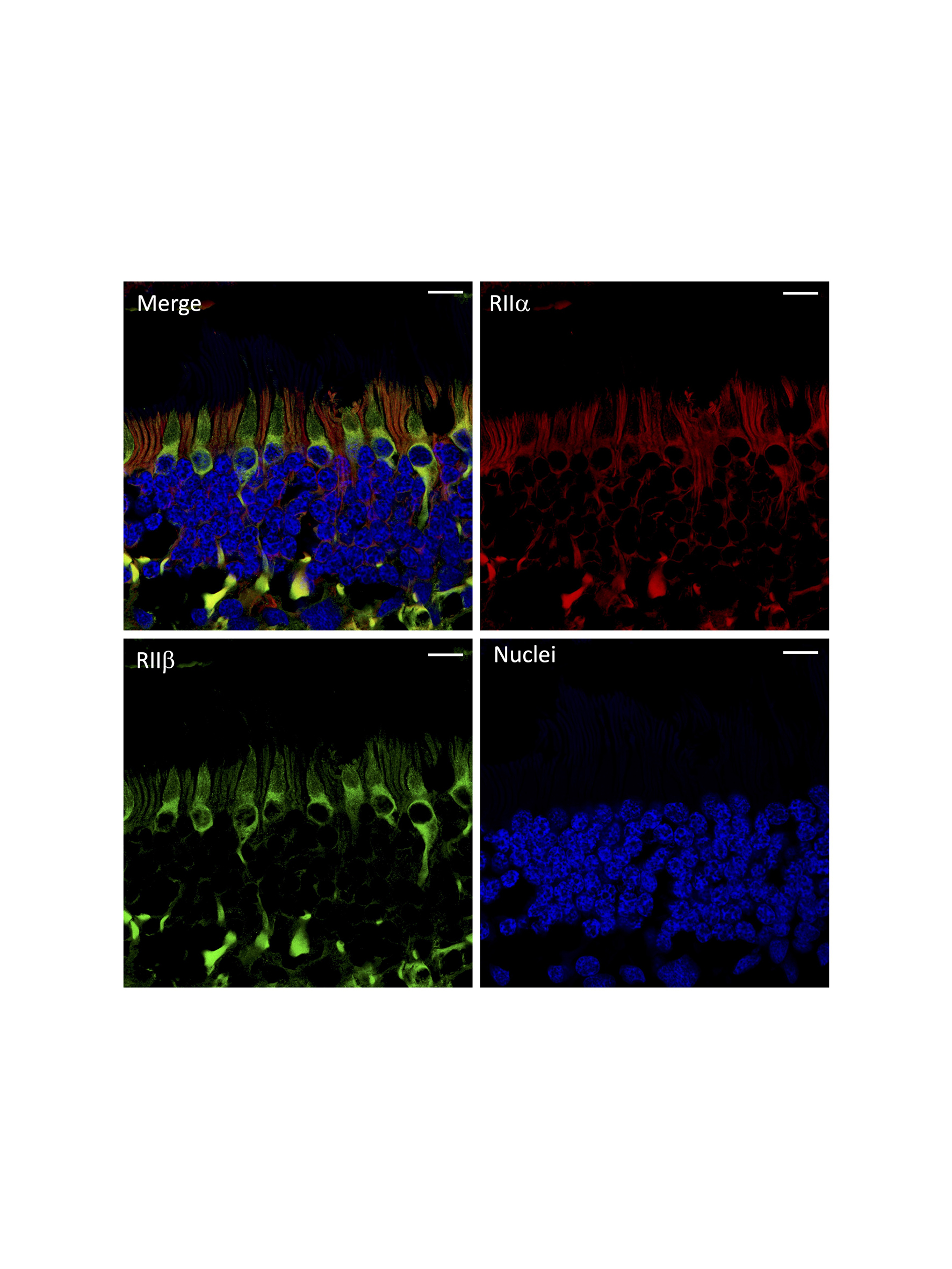

Supplement: Supplementary Figure 4 — Airyscan images of RIIβ + RIIα in photoreceptor cells. Z-stack Airyscan images confirm intracellular localization of RIIβ (green, RIIβ) and RIIα (red, RIIα), with clear co-localization of RIIβ and RIIα (yellow, Merge) in the cell body and axon of cone cells. Nuclei in blue, scale bar = 10 mm. [file Image_4.JPEG]

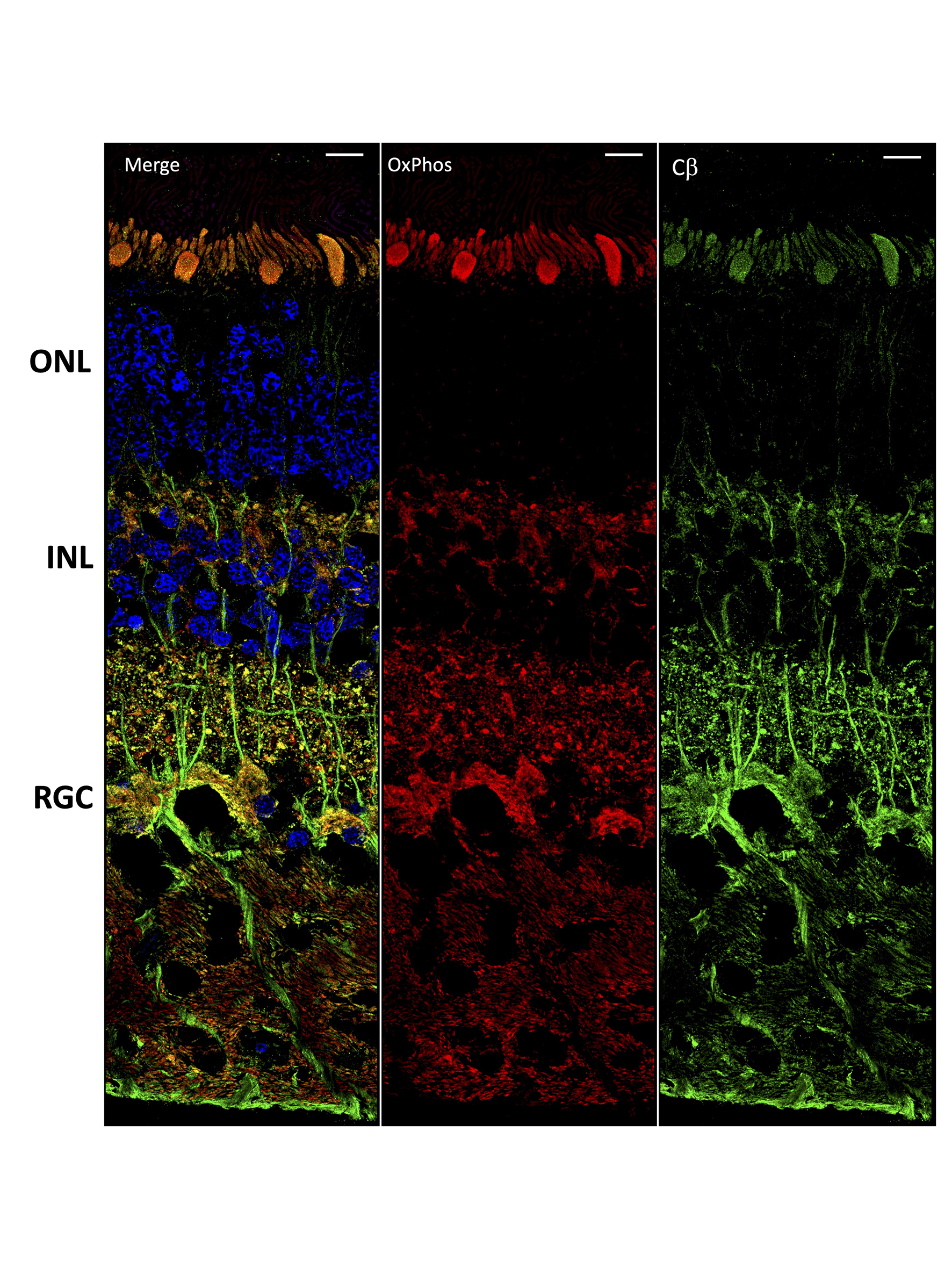

Supplement: Supplementary Figure 5 — Airyscan images of Cβ + mitochondria. Cb (green) and OxPhos(red)-labeled mitochondria co-localize in the ONL, INL, and RGC, with clear signal overlap (yellow) in every cell layer. Nuclei in blue, scale bar = 10 mm. [file Image_5.JPEG]

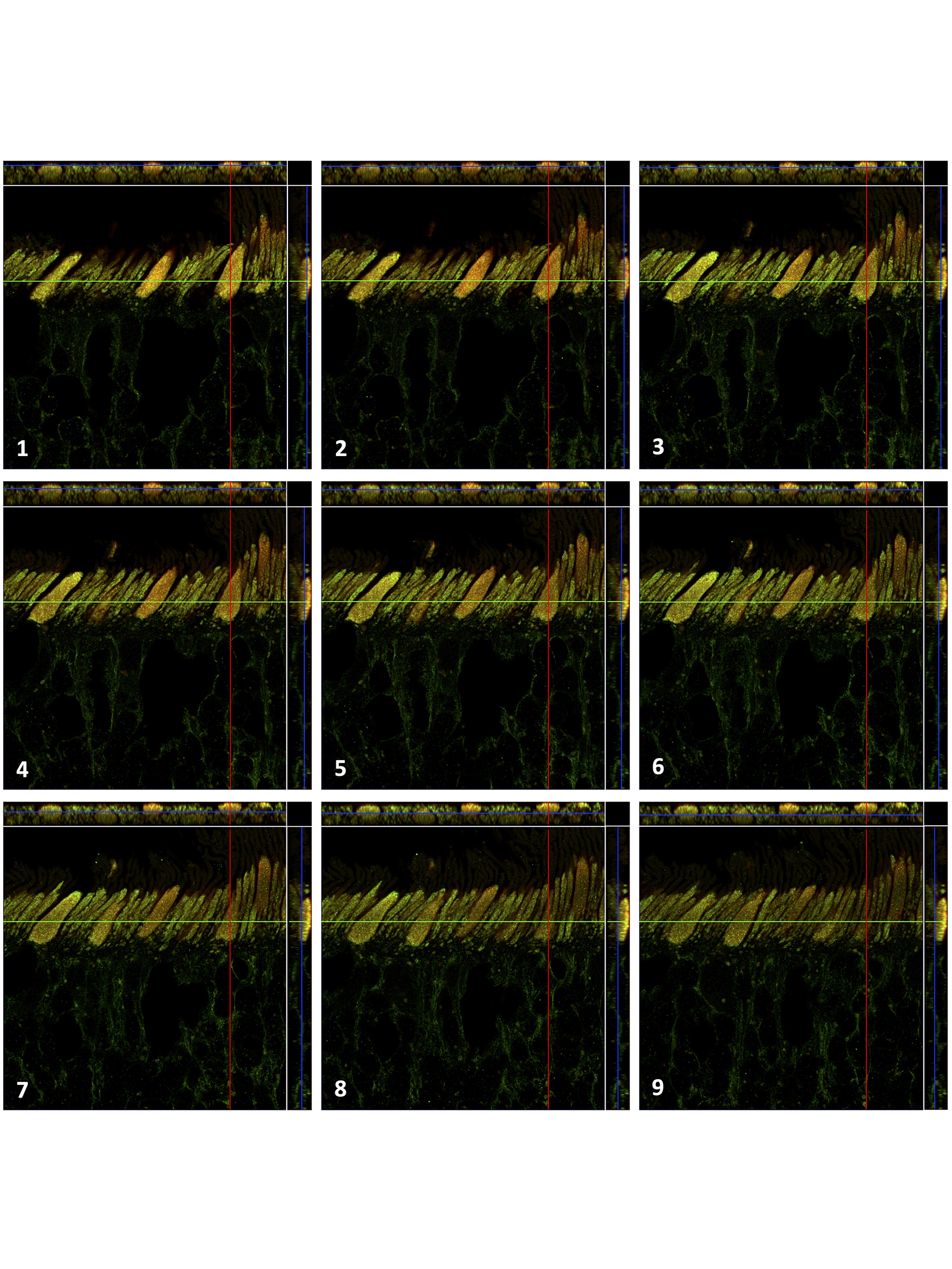

Supplement: Supplementary Figure 6 — Z-stack images of Cβ + mitochondria. Nine z-stack Airyscan images highlight intracellular localization of Cβ and mitochondria co-localization (yellow). As the panels move from image 1 (top stack, blue line) to image 9 (bottom stack, blue line) it is clear that Cβ is continuously co-localized mitochondria in throughout the entire photoreceptor inner segment. Blue line = z position (primary image), green line = top panel, red line = right panel. Green and red lines highlight localization in an individual cell, which can be further visualized in the top and right panel. [file Image_6.JPEG]

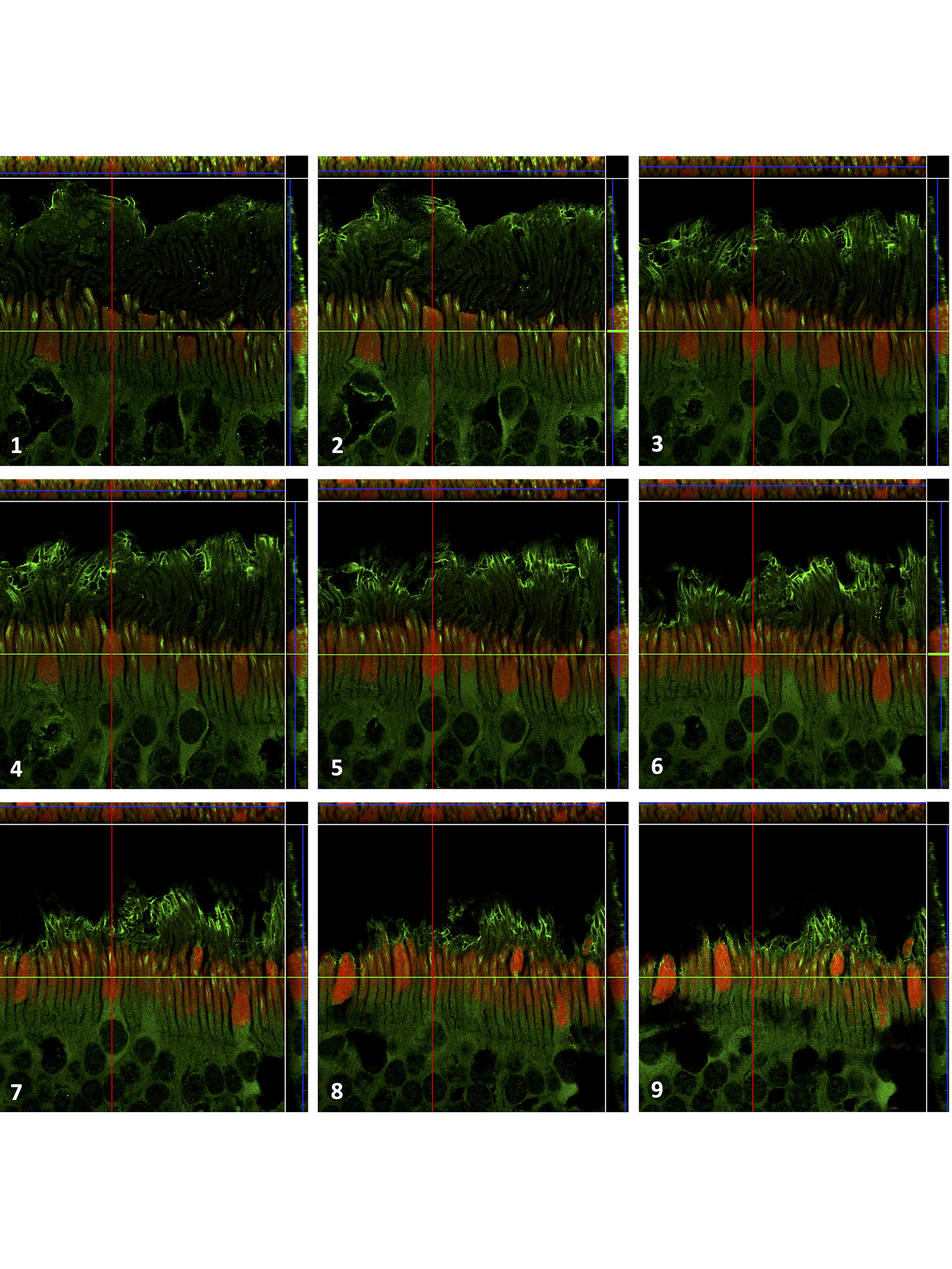

Supplement: Supplementary Figure 7 — Z-stack images of Cα + mitochondria. Nine z-stack Airyscan images highlight intracellular localization of Cα (green) with respect to mitochondria (red). As the panels move from image 1 (bottom stack, blue line) to image 9 (top stack, blue line) it is clear that Cα is continuously expressed in the outer segment membrane. There is clear signal from Cα (green) at the top of the outer segment in images 1–3, in the mid region in images 4–6, and near the base (closer to the inner segment mitochondria ellipsoid) in images 7–9. Images 1–9 also confirm Cα (green) is not present in the mitochondria ellipsoid (red). Blue line = z position (primary image), green line = top panel, red line = right panel. Green and red lines highlight localization in an individual cell, which can be further visualized in the top and right panel. [file Image_7.JPEG]

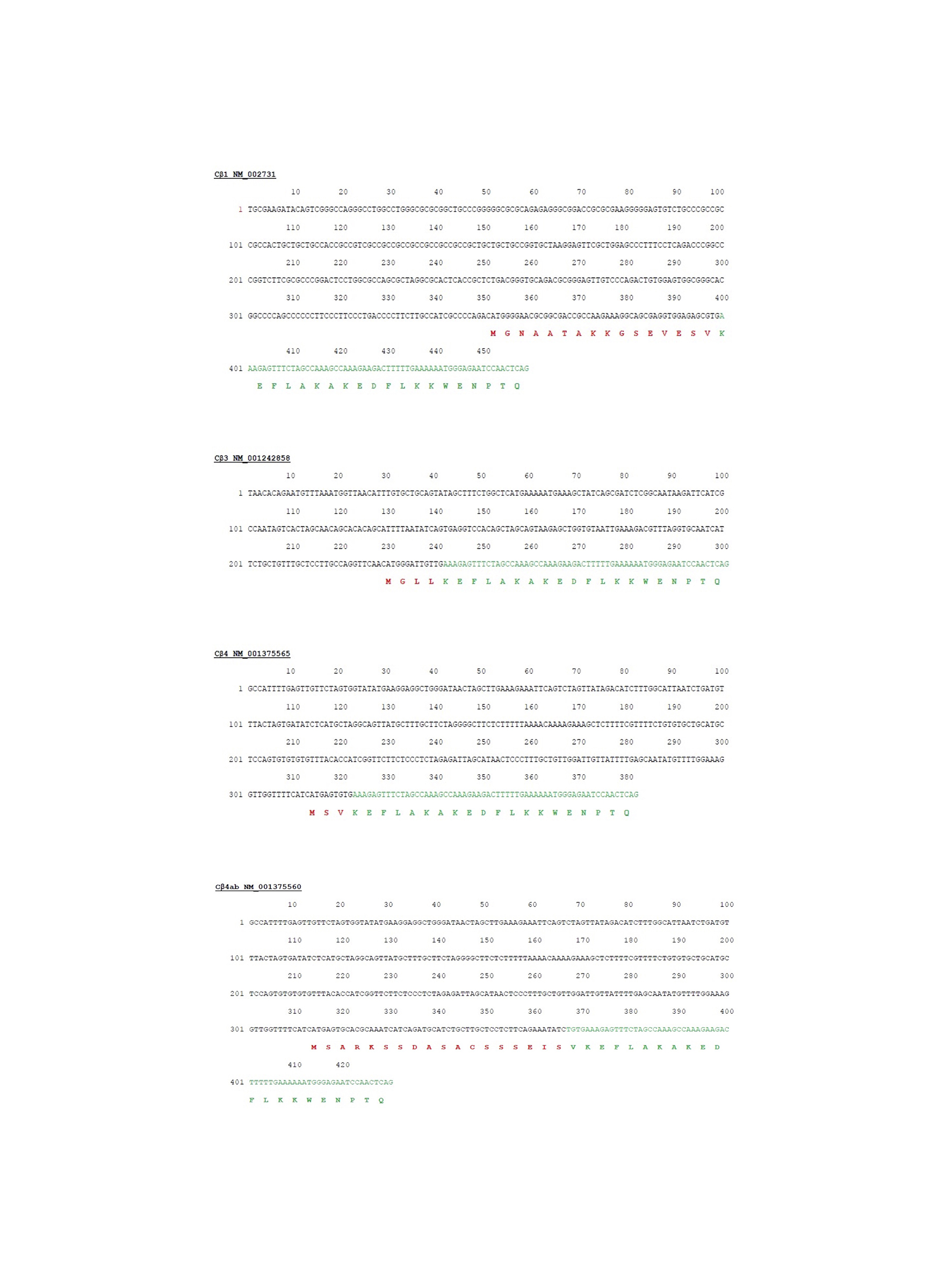

Supplement: Supplementary Figure 8 — RNA sequence alignments for Cβ1, Cβ3, Cβ4, and Cβ4ab. Differences at the N-terminus were sufficient to design BaseScope probes specific for each isoform (red section), with sequence similarities continuing beyond exon 1 (green section). [file Image_8.JPEG]

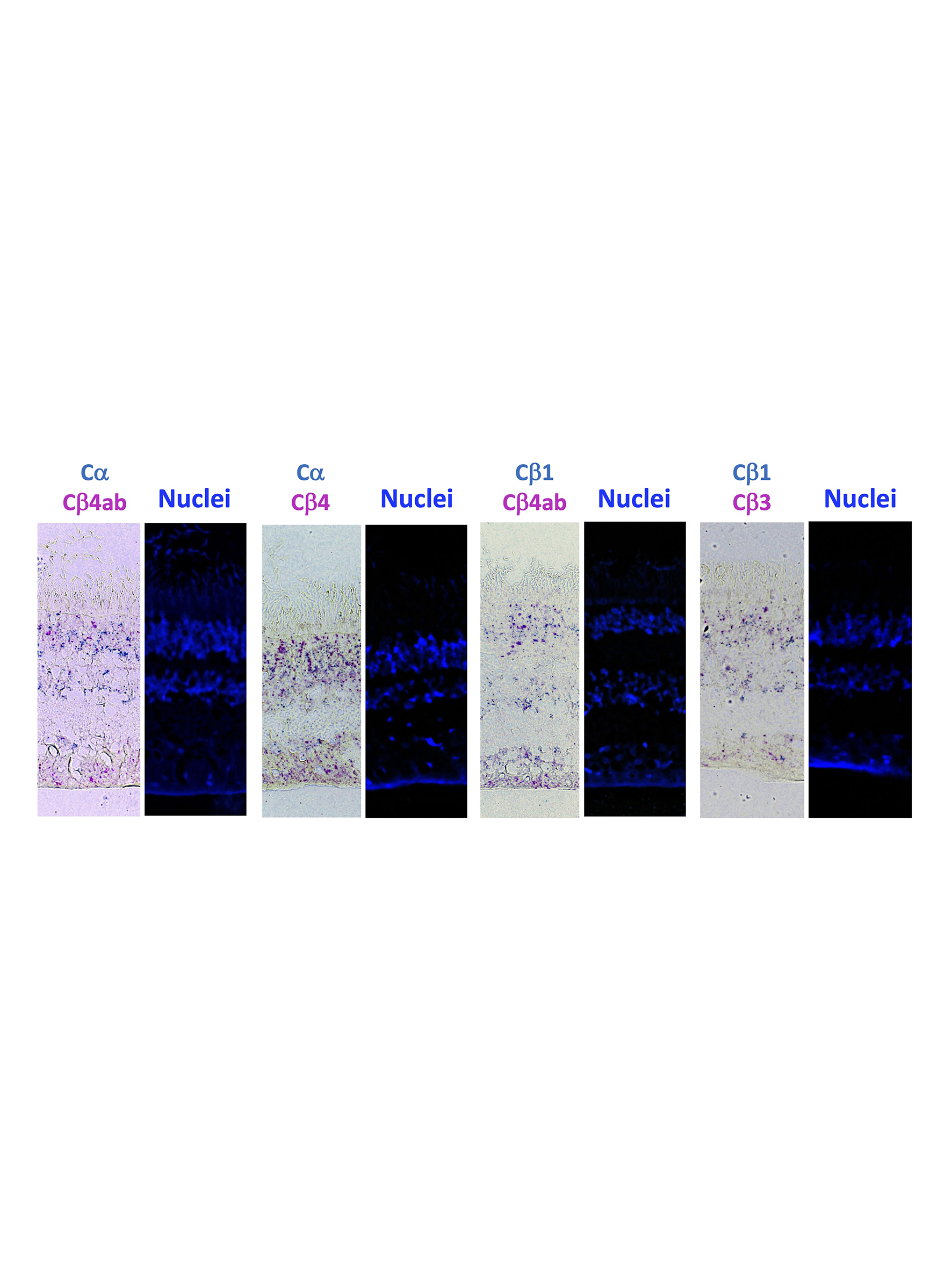

Supplement: Supplementary Figure 9 — BaseScope Duplex assay of Cα, Cβ1, Cβ3, Cβ4, and Cβ4ab expression. All isoforms are expressed in photoreceptor and interneuron cells, with Cβ4 and Cβ4ab prominently expressed in all tissue layers. Color deconvolution was done following the technical note of ACD (TS 46-003/RevA/Date 6212018). The green color and red color were separated with Image J, darkened, and merged together using Adobe Photoshop. [file Image_9.JPEG]

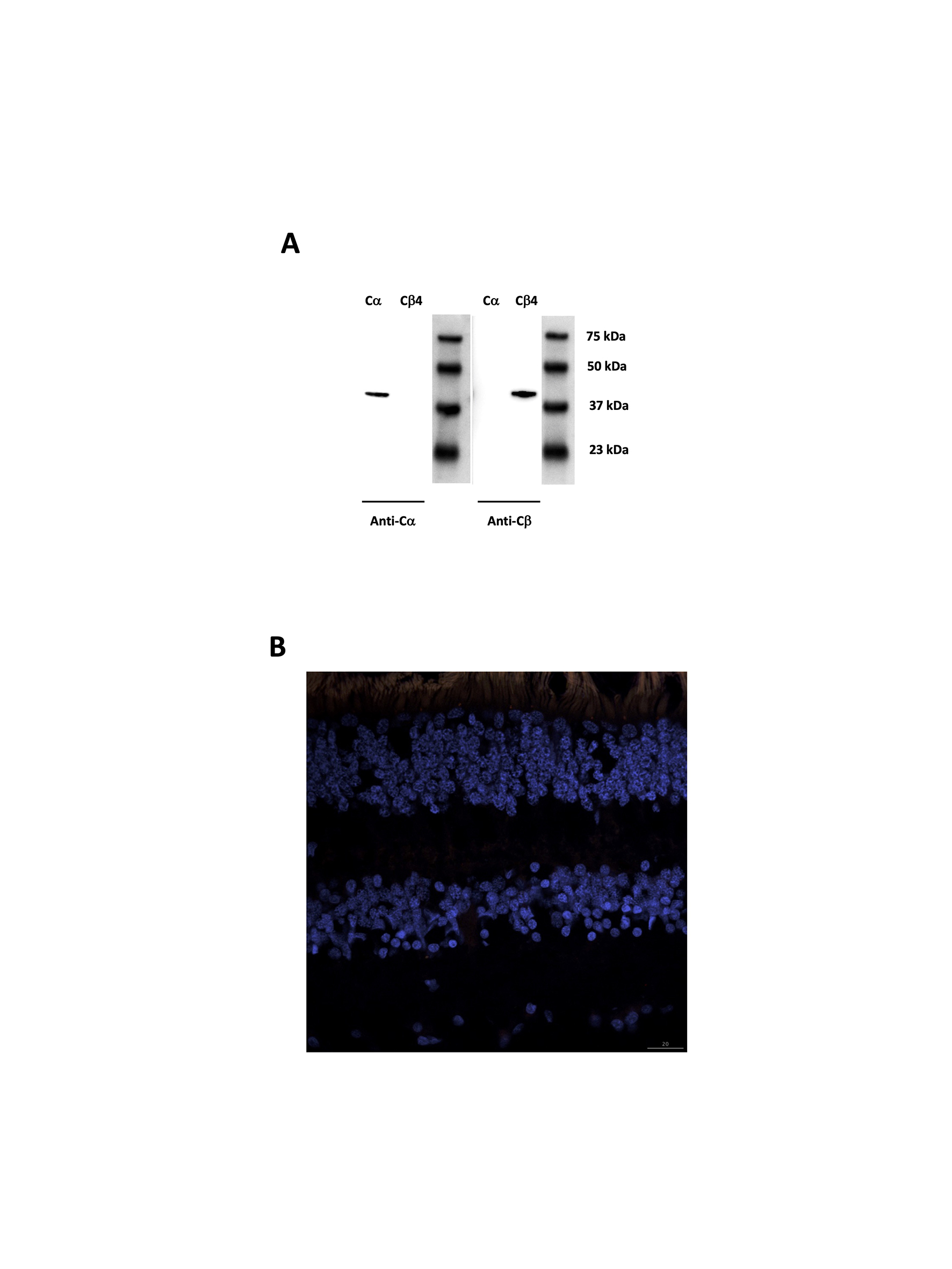

Supplement: Supplementary Figure 10 — Antibody control for Cα and Cβ. (A) Anti-Cα antibodies specifically recognized purified Cα protein and not purified Cβ4 protein in Western blots, while anti-Cβ antibodies recognized purified Cα protein and not purified Cβ4 protein. Both Cα and Cβ4 were present at the predicted size of ∼39 kDa. (B) Human retina sections used as negative controls for corresponding IHC data showed no detectable signal after incubation with secondary donkey anti-rabbit and donkey anti-mouse antibodies. [file Image_10.JPEG]
